# Infants exposed *in utero* to Hurricane Maria have gut microbiomes with reduced diversity and altered metabolic capacity

**DOI:** 10.1128/msphere.00134-23

**Published:** 2023-09-27

**Authors:** Ai Zhang, David de Ángel Solá, Midnela Acevedo Flores, Lijuan Cao, Leran Wang, Josh G. Kim, Phillip I. Tarr, Barbara B. Warner, Nicolás Rosario Matos, Leyao Wang

**Affiliations:** 1 Department of Medicine, Division of Allergy and Immunology, Washington University School of Medicine in St. Louis, St. Louis, Missouri, USA; 2 Department of Pediatrics, Yale University School of Medicine, New Haven, Connecticut, USA; 3 Department of Pediatrics and Obstetrics and Gynecology, San Juan City Hospital Research Unit, San Juan Hospital, San Juan, Puerto Rico; 4 Department of Medicine, Division of Infectious Diseases, Edison Family Center for Genome Sciences and Systems Biology, Washington University School of Medicine in St. Louis, St. Louis, Missouri, USA; 5 Department of Pediatrics, Washington University School of Medicine in St. Louis, St. Louis, Missouri, USA; Nanjing University of Chinese Medicine, Nanjing, Jiangsu, China

**Keywords:** infant gut microbiome, prenatal exposure, extreme weather event, climate change, metagenomic sequencing, asthma

## Abstract

The gut microbiome is a potentially important mechanism that links prenatal disaster exposures with increased disease risks. However, whether prenatal disaster exposures are associated with alterations in the infant’s gut microbiome remains unknown. We established a birth cohort study named Hurricane as the Origin of Later Alterations in Microbiome (HOLA) after Hurricane Maria struck Puerto Rico in 2017. We enrolled vaginally born Latino term infants aged 2 to 6 months, including *n* = 29 infants who were exposed *in utero* to Hurricane Maria in Puerto Rico and *n* = 34 infants who were conceived at least 5 months after the hurricane as controls. Shotgun metagenomic sequencing was performed on infant stool swabs. Infants exposed *in utero* to Hurricane Maria had a reduced diversity in their gut microbiome compared to the control infants, which was mainly seen in the exclusively formula-fed group (*P* = 0.02). Four bacterial species, including *Bacteroides vulgatus*, *Clostridium innocuum*, *Bifidobacterium pseudocatenulatum*, and *Clostridium neonatale*, were depleted in the exposure group compared to the control group. Compositional differences in the microbial community and metabolic genes between the exposure and control groups were significant, which were driven by the formula feeding group (*P* = 0.02 for the microbial community and *P* = 0.008 for the metabolic genes). Metabolic modules involved in carbohydrate metabolism were reduced in the exposure group. Prenatal maternal exposure to Hurricane Maria was associated with a reduced gut commensal and an altered microbial composition and metabolic potential in the offspring’s gut. Breastfeeding can adjust the composition of the gut microbiomes of exposed infants.

**IMPORTANCE:**

Climate change is a serious issue that is affecting human health. With more frequent and intense weather disasters due to climate change, there is an urgent need to evaluate and understand the impacts of prenatal disaster exposures on the offspring. The prenatal stage is a particularly vulnerable stage for disease origination. However, the impact of prenatal weather disaster exposures on the offspring’s gut microbiome has not been evaluated. Our HOLA study starts to fill this knowledge gap and provides novel insights into the microbiome as a mechanism that links prenatal disaster exposures with elevated disease risks. Our major finding that reduced microbial diversity and altered metabolic capacity are associated with prenatal hurricane exposures warrants further studies to evaluate the impact of weather disasters on the unborn.

## INTRODUCTION

Prenatal adverse exposures increase the risk of developing a variety of adverse health conditions (1 – 6). A natural disaster can trigger a complex mix of acute psychological, physiological, and environmental stress (7 – 10). Such exposures in pregnant women can affect the developing fetus (11 – 14). The combination of these adverse exposures during the prenatal stage has more profound effects on the offspring’s development compared to a single exposure (15). Since climate change has increased the frequency and intensity of weather disasters, there is an urgent need to understand the impacts of *in utero* weather-disaster exposures on the offspring (16, 17). Insights into the link between maternal exposures to a disaster and elevated disease risks in the offspring are needed to synthesize a comprehensive network map of this public health issue. This data will lead to the development of evidence-based disease prevention strategies and target groups that are exposed to disasters during their critical prenatal stage (12, 18, 19).

The early human microbiome is a potential mechanism that links prenatal weather disaster exposures to adverse health outcomes, but studies on this link have been limited (9, 20). Mounting evidence suggests that bacterial colonizers in the intestines are critical in developing an infant’s immune system (21, 22). Microbe-immune interactions are the primary driver of an ordered immune imprinting sequence in early infancy (21). The aberrant microbial composition and ecological function in the infant gut have been implicated in immune dysregulation and metabolic and psychological disorders (23 – 25). During early life, the infant gut microbiome can be affected by several maternal and lifestyle factors, such as birth mode, breastfeeding, and antibiotic use (26, 27). Prenatal adverse exposures, including maternal psychological stress and pollutant exposures, can also disrupt the development of an offspring’s gut microbiome (28 – 32). This suggests that prenatal disaster exposures may be associated with an altered gut microbiome in infancy, which may be an important mechanism that leads to increased disease risks throughout the life cycle.

To investigate this association, we established a birth cohort study named Hurricane as the Origin of Later Alterations in Microbiome (HOLA) after Hurricane Maria, a Category 4 storm, struck Puerto Rico on September 20, 2017. It was one of the deadliest natural disasters in the United States, with an estimated 4,645 excess deaths and more than $90 billion in damages (33, 34). Puerto Ricans experienced negative health impacts and post-traumatic stress due to a combination of physical and psychological challenges, including hazardous exposures, economic hardship, and prolonged and extensive disruptions in electricity, water supply, communication, transportation, and medical services (35 – 37). Pregnant women were a vulnerable population and endured widespread structural damage in their homes, insufficient healthcare access, nutritional food shortages, and increased exposures to pollutants (35, 38). Our HOLA study recruited 29 infants who were exposed *in utero* to Hurricane Maria and 34 infants who were conceived at least 5 months after the hurricane in the control group. We performed shotgun metagenomic sequencing on their stool swab samples and compared differences in the microbial compositions and metabolic potentials.

## MATERIALS AND METHODS

### Participant recruitment, questionnaire, and stool sample collection

Our HOLA study was reviewed and approved by the Institutional Review Board of San Juan City Hospital (ID: EPDC-Microbiome). This study was deemed not related to human subjects by the Washington University Human Research Protection Office because all the data and samples were de-identified when they were received at Washington University. The San Juan City Hospital Research Unit recruited and enrolled 29 infants in the disaster exposure group between April and August 2018 and 34 infants in the control group between March and September 2019. Infants in the exposure group were *in utero* when Hurricane Maria struck Puerto Rico, and those in the control group were *in utero* starting at least 5 months after Hurricane Maria. Inclusion criteria for both groups included the following: (i) full-term birth (at least 36 weeks of gestational age), (ii) birth by a vaginal delivery, (iii) aged between 2 and 6 months, (iv) residence in Puerto Rico throughout the mother’s pregnancy, and (v) currently not receiving any medications. Exclusion criteria for both groups were the following: (i) delivery by a cesarean section, (ii) premature birth (less than 36 weeks of gestational age), (iii) admission to a neonatal intensive care unit, (iv) acute illness at the time of sample acquisition, (v) airway or pulmonary malformations, (vi) identified chromosomal or genetic abnormalities, and (vii) low birthweight (less than 2,500 grams). All the parents signed a written informed consent to participate in this study. Every mother completed a Spanish-language questionnaire on-site at the Research Unit. Questions about infant feeding referred to the timeframe before sample collection. Questions about exposures and experiences during the aftermath of Hurricane Maria were developed based on various sources, including instruments used at the time by psychology researchers on the island and the personal disaster experiences of research team members, particularly those who lived in Puerto Rico or were actively involved in disaster relief immediately after the hurricane. These questions asked about the fear of injuries and deaths, family injuries and losses, health problems, limited prenatal care, displacement, access to electric power, environmental exposures, access to drinking water, food, or medication, and changes in family income. The questionnaire was subsequently translated into English and listed as a supplementary file in our previous paper (9). The questionnaire also included the Edinburgh Postnatal Depression Scale (EPDS), a standard psychological instrument that detects depressive symptoms (39). During analysis, two researchers independently extracted data from the questionnaires and calculated an EPDS score. Any discrepancies between the scores calculated by the two researchers were resolved through discussions.

Fresh stool samples were collected directly from each infant’s diaper liner with a sterile swab at the clinic or during a home visit by a trained health professional. The swab was immediately placed into a DNA/RNA Shield collection solution (Zymo Research, Irvine, CA, USA) and shipped to Washington University for bacterial DNA extraction. Every DNA/RNA Shield collection tube contained DNA and RNA stabilization solutions, which preserved the integrity of the nucleic acids in the samples. All samples in the collection tubes remained at room temperature during storage and transportation, per the manufacturer’s instructions.

### DNA extraction and metagenomic sequencing

Microbial DNA was extracted from each sample using a ZymoBIOMICS DNA Miniprep Kit (Zymo Research). Shotgun metagenomic sequencing was performed at Washington University GTAC@MGI. The extracted DNA (100 to 250 ng) was fragmented on the Covaris LE220, targeting 375 bp inserts. Automated dual-indexed libraries were constructed using the KAPA Hyper Library Prep Kit (Roche) on the SciClone NGS instrument (Perkin Elmer). The ligated fragments were amplified with KAPA HiFi ReadyMix (Roche) and 8 cycles of polymerase chain reaction (PCR). The amplified libraries were size-selected with a dual-spiral AMPure bead purification, targeting an average final library size of 450 bp. The libraries were assessed on the Caliper GX (Perkin Elmer) to determine their size and mass. Libraries were diluted to 5 nM, and the concentration of each library was validated by performing qPCR using the KAPA Library Quantification Kit (Roche) and the Lightcycler 480 (Roche). Libraries were normalized to 2.5 nM and analyzed using the NovaSeq 6000 S4 300 cycle kit and the XP workflow (Illumina). Approximately 12 GB of 2× 150 paired-end sequence data were generated per sample.

### Sequencing data analysis

Raw paired-end sequencing reads were quality-controlled with KneadData version 0.10.0 (Anon., n.d.). This involved the removal of TruSeq library adaptors and low-quality reads using Trimmomatic version 0.39 with these parameters: LEADING:3, TRAILING:3, SLIDINGWINDOW:4:15, and MINLEN:36. This was followed by the identification and removal of host (human) reads by mapping against the human genome (hg37 build) with Bowtie 2 version 2.4.2 in paired-end mode. Taxonomic profiling and quantification of quality-filtered metagenomes were performed using the Metagenomic Phylogenic Analysis (MetaPhlAn3, version 3.0.7) with default parameters (40). Briefly, MetaPhlAn3 estimated microbial relative abundances by mapping metagenomic reads against a database of clade-specific marker sequences spanning bacterial, eukaryotic, and archaeal phylogenies. Quality-filtered metagenomic samples were functionally profiled using the HMP Unified Metabolic Analysis Network (HUMAnN3) version 3.0.0. in UniRef90 mode with default parameters (40). HUMAnN3 initially identified known microbial species in a sample using MetaPhlAn3 and constructed a sample-specific database by merging functionally annotated pangenomes of the identified species. Then, HUMAnN3 mapped the sample reads against that sample’s pangenome database using Bowtie2 (41). Sample reads that failed to align with the sample’s pangenome database were mapped to UniRef90 by an accelerated translated search with DIAMOND (42). The cladograms were generated with the output file from MetaPhlAn3 using GraPhlan. For this study, gene family output was first regrouped to Kyoto Encyclopedia of Genes and Genomes (KEGG) orthogroups with utility_mapping/map_ko_uniref90.txt.gz, and then the humann_legacy-0.99b/data/modulec map to KEGG modules was used (43). The KEGG module abundances were normalized to counts per million.

### Statistical analysis

All statistical analyses were conducted in R version 4.1.1. Alpha diversity was determined based on species-level abundance using the estimate_richness() function in the phyloseq package version 1.38.0. Weighted UniFrac distances were generated using the unifrac() function in rbiom version 1.0.3 with the Newick tree metaphlan/utils/mpa_v30_CHOCOPhlAn_201901_species_tree.nwk. Three species (*Collinsella stercoris*, *Granulicatella elegans*, and *Sutterella parvirubra*) were not present in the tree file and were not included in this calculation. Principal coordinate decompositions were computed using the pcoa() function in the ape package version 5.6-2. Distance matrices were used to perform principal coordinate analysis (PcoA) using the anosim() function in the vegan package version 2.6-2. Wilcoxon rank sum tests were used to test the significance of continuous variables of characteristics and alpha diversity by groups. The significant difference in gene family counts between the groups was tested using the Student’s *t*-test. Chi-squared tests were used to calculate the significance of categorical variables and characteristics. Fisher’s exact tests were conducted to calculate the significant difference in the presence of species by groups in each feeding type. Microbiome Multivariable Association with Linear Models (MaAsLin2) was used to identify bacterial species or KEGG modules that were associated with prenatal hurricane exposure (44). Box plots, bar plots, and PCoA plots were generated using the ggplot2 package version 3.3.5. Heatmaps were generated using the heatmap package version 1.0.12. *P*-values and multiple testing were adjusted using the false discovery rate method.

## RESULTS

### Characterization of the HOLA cohort and microbial community

We recruited a total of 63 infants in our HOLA study, including 29 infants in the exposure group and 34 infants in the control group. Infants were recruited from the metropolitan region of San Juan. The infants received medical care from satellite clinics and local health facilities that primarily serve low-income patients with medical insurance provided by the state. The participants' characteristics are presented in Table 1. All infants were Latino and born vaginally and full-term. There were no significant differences between the exposure and control groups with respect to the infants’ age at the time of sampling, the presence of siblings living in the household, feeding types, or the mothers’ risk for depression indicated by the EPDS score. There were more male infants in the control group than in the exposure group, and this difference had borderline significance (*P* = 0.054). All mothers reported power outages and exposures to fumes, mold, or debris during their pregnancy due to Hurricane Maria and its aftermath.

**TABLE 1:** Characteristics of the study participants^*a*^

Variables	Exposure group (*n* = 29)	Control group (*n* = 34)	*P*
Infant sex: male, *n* (%)	10 (34.48)	20 (58.82)	0.054
Infant ethnicity: Latino, *n* (%)	29 (100)	34 (100)	–^*d*^
Infant age (weeks), mean (SD)^*b*^	15.61 (5.52)	16.36 (4.39)	0.34
Gestational age in weeks, mean (SD)	38.86 (1.00)	38.83 (1.36)	0.80
Presence of siblings at home, *n* (%)	19 (65.52)	24 (70.59)	0.67
Exclusive breastfeeding, *n* (%)	8 (27.59)	10 (29.41)	0.87
Maternal EPDS^*c*^ score, mean (SD)	5.45 (4.85)	4.68 (3.76)	0.72
Antibiotic use in the past 4 weeks	3 (10.34)	1 (2.94)	0.23
Trimester in pregnancy on the day of Hurricane Maria			
First, *n* (%)	8 (27.59)	–	–
Second, *n* (%)	21 (72.41)	–	–
Feeding type			0.086
Exclusive breast milk, *n* (%)	8 (27.59)	10 (29.41)	
Mixed, *n* (%)	13 (44.83)	7 (20.59)	
Formula, *n* (%)	8 (27.59)	17 (50.00)	

^*a*^ Significance was evaluated based on the Wilcoxon rank sum test and the Chi-squared test.

^*b*^ SD, standard deviation.

^*c*^ EPDS, Edinburgh Postnatal Depression Scale.

^*d*^ –, not applicable.

To characterize the gut microbiome, shotgun metagenomic sequencing was performed on DNA extracted from stool swabs. We obtained a total of 5,361,718,902 raw sequencing reads. After quality control procedures, 3,977,710,847 high-quality reads were retained for downstream analysis (Fig. S1A). The exposure group had 1,870,919,014 high-quality reads for 29 samples, and the mean read number was 64,514,449. The control group had 2,106,791,833 high-quality reads for 34 samples, and the mean read number was 61,964,466. The number of sequencing reads from each sample was adequate to determine the number of species based on the rarefaction curve (Fig. S1B).

**FIG 1:**
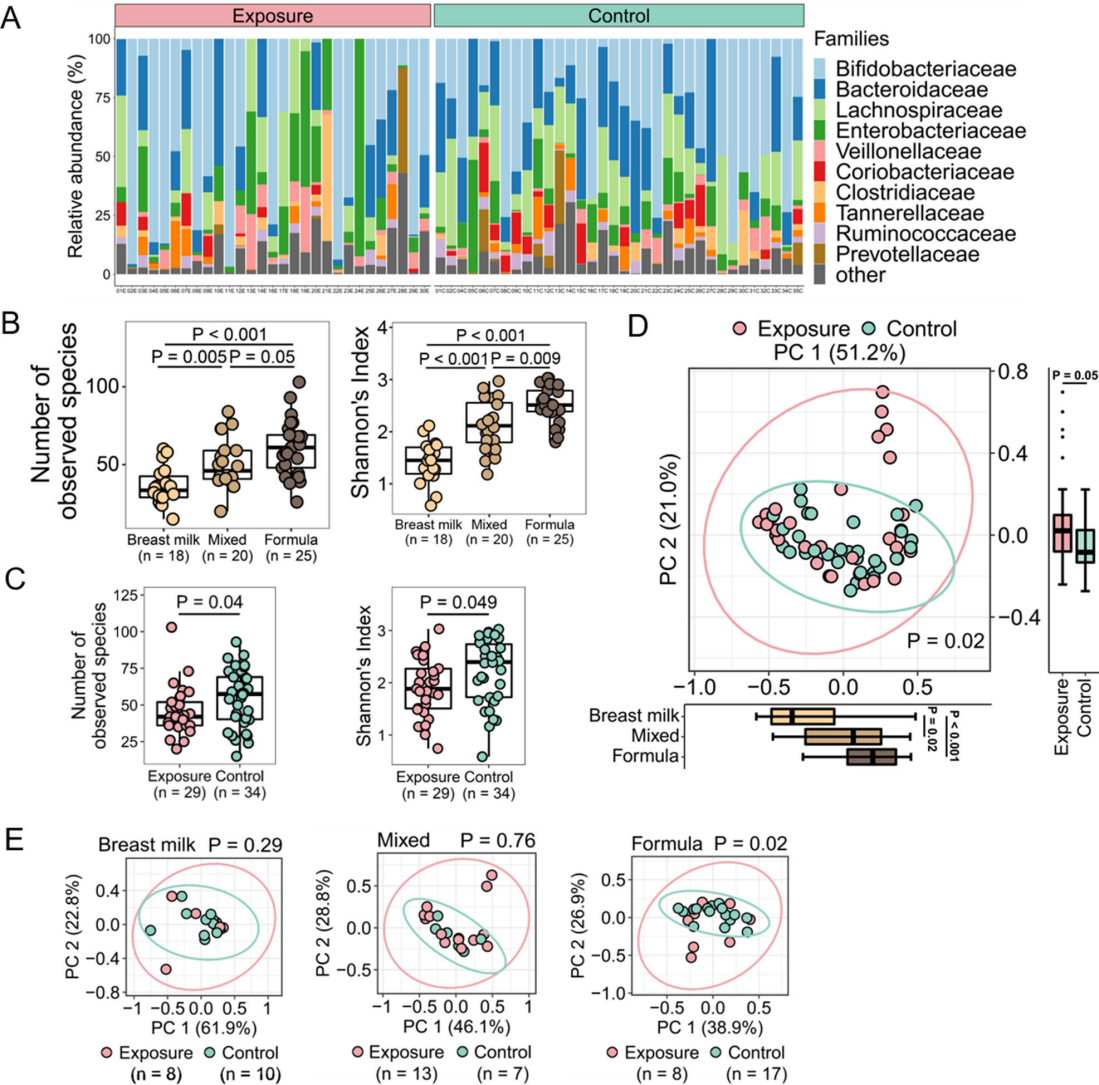
Characterization of the infant gut microbiome at the community level. (A) Profile of the top 10 most abundant bacterial families in the infant gut microbiome of each sample. Each bar represents the gut bacterial community of an infant and the relative abundance of each family. (B) Analysis of α-diversity by the feeding type. Left, comparison of the number of species between each pair of feeding types; right, comparison of the community richness and evenness represented by the Shannon index between each pair of feeding types. (C) Analysis of α-diversity by the prenatal disaster exposure status. Left, comparison of the number of species between the exposure and control groups; right, comparison of the community richness and evenness represented by the Shannon index between the exposure and control groups. (D) Principal coordinate analysis of β-diversity by the prenatal disaster exposure status based on weighted UniFrac distances. (E) Principal coordinate analysis of β-diversity by the prenatal disaster exposure status for each feeding type based on weighted UniFrac distances. Statistical significance in (B) and (C) was determined using the Wilcoxon rank sum test. Statistical significance in (D) and (E) was determined using the analysis of similarities test. PC, principal coordinate.

A total of 398 species were detected in all 63 stool swab samples. There were 227 species that appeared in both groups and accounted for most of the observed taxa (98.2% across all samples). There were 67 species that only appeared in the exposure group (accounting for 0.9% of the total reads) and 104 species that only appeared in the control group (accounting for 0.9% of the total reads). The top four most abundant bacterial phyla corresponded to more than 99% of all reads, including Actinobacteria (33.69%), Firmicutes (23.31%), Bacteroidetes (28.34%), and Proteobacteria (14.12%). There were no significant differences in the relative abundances of these bacterial phyla between the exposure and control groups. The top 10 most abundant bacterial families across all samples were plotted for each sample (Fig. 1A) and included Bifidobacteriaceae (31.27%), Bacteroidaceae (24.10%), Lachnospiraceae (11.77%), Enterobacteriaceae (13.42%), Veillonellaceae (2.16%), Coriobacteriaceae (1.90%), Clostridiaceae (3.28%), Tannerellaceae (2.91%), Ruminococcaceae (2.15%), and Prevotellaceae (1.05%).

### Community-level differences in the infant gut microbiome

We first evaluated factors at the community level that might have affected the infant gut microbiome. Results indicated that the infant feeding type, including exclusive breastfeeding, mixed breastfeeding and formula feeding, and exclusive formula feeding, was significantly associated with the infant gut microbiome (Fig. 1B). Breastfed infants had the lowest number of bacterial species among the three feeding types (*P* < 0.001 for breastmilk versus (vs) formula; *P* = 0.005 for breastmilk vs mixed). Infants who were fed a mix of breastmilk and formula had fewer bacterial species than formula-fed infants (*P* = 0.055). The Shannon index, which characterized the richness and evenness of the gut microbial community, detected these same trends (*P* < 0.001 for breastmilk vs formula; *P* = 0.001 for breastmilk vs mixed; *P* = 0.009 for mixed vs formula), which were also consistent with those from previous studies (45, 46). These associations were also observed in the exposure and control groups, respectively (Fig. S2). We examined several other factors, including infant sex, antibiotic use in the previous 4 weeks, the presence of siblings at home, infant age at sampling, and maternal EPDS score at sampling, but we did not observe a significant correlation between these factors and the infant gut microbiome at the community level (Fig. S3).

We then analyzed whether infants in the exposure group harbored a different gut microbiome than infants in the control group. The number of bacterial species and Shannon index were significantly lower in the exposure group than in the control group, indicating a reduced diversity in the gut microbial community in infants who were exposed *in utero* to the hurricane (Fig. 1C). Through a subgroup analysis, we also examined whether differences in microbial diversity were confounded by the feeding type. There were no differences between the exposure and control groups in the number of bacterial species or Shannon index in infants who were breastfed. In contrast, we observed similar trends of lower bacterial diversity in infants fed with formula or mixed breastmilk plus formula in the exposure group, although it was only statistically significant in infants under the mixed feeding type (Fig. S4).

The gut microbial composition also significantly differed between the exposure and control groups (*P* = 0.02), with the feeding type accounting for significant differences in the top principal coordinate and the exposure status accounting for significant differences in the second top principal coordinate (Fig. 1D). We compared the microbial differences for each feeding type and found differences in infants fed with formula (*P* = 0.02, Fig. 1E). In summary, at the community level, prenatal exposure to Hurricane Maria was associated with reduced diversity and an altered composition in the infant gut microbiome. The microbial compositional difference was primarily observed in infants who were exclusively fed formula.

### Species-level differences in the infant gut microbiome

**FIG 2:**
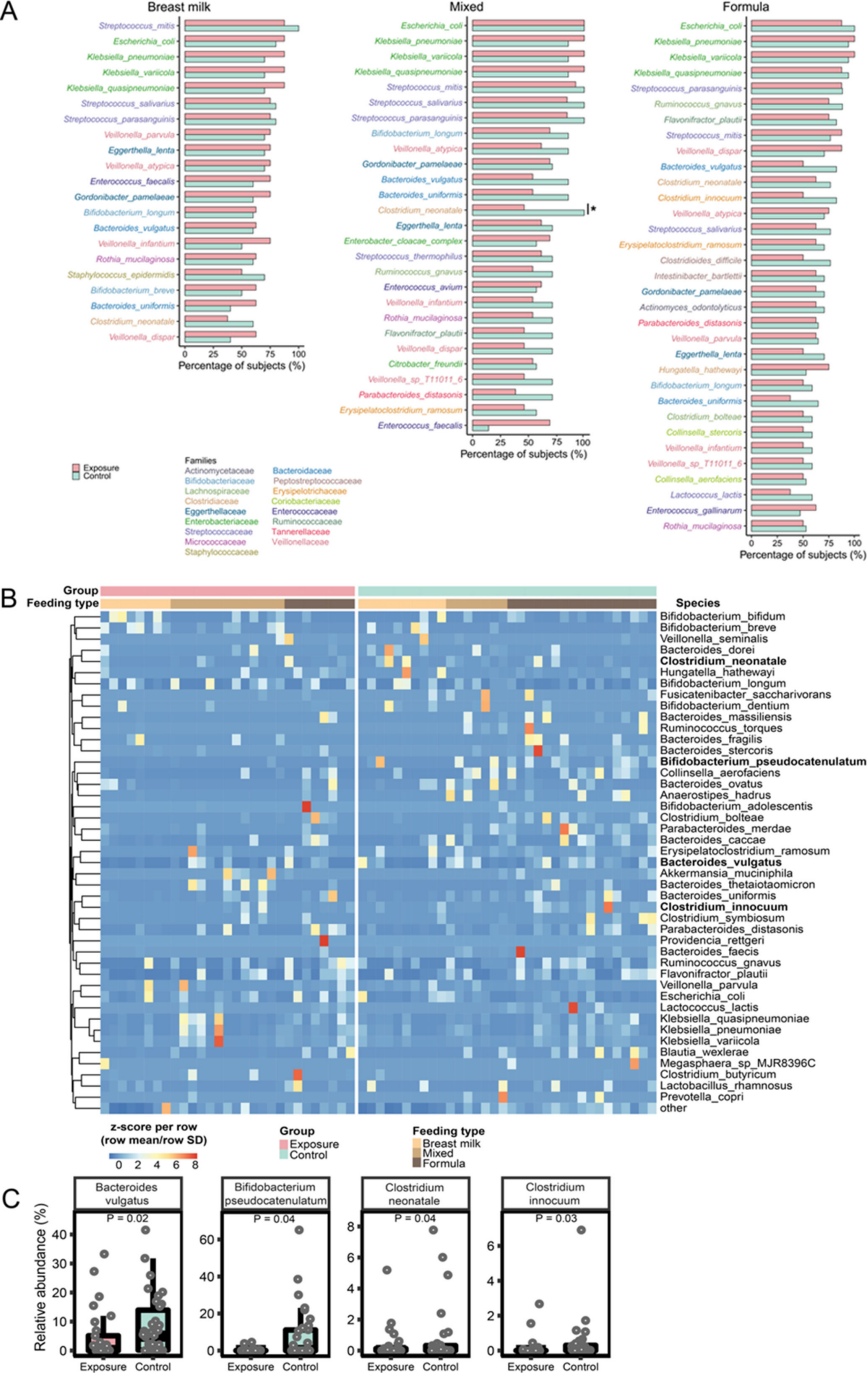
Species-level comparisons of the gut microbiome in the exposure and control groups. (A) Species with a prevalence greater than 50% for each feeding type were compared in the exposure and control groups (prevalence expressed as percentage). Statistical significance was evaluated based on the Fisher’s exact test. (B) Heatmap displaying the relative abundance (*z*-score of normalized relative abundance for each row) of the 44 most abundant bacterial species. Each column represents a sample grouped by its exposure status (exposure or control), feeding type, and infant age at sampling, from youngest to oldest. Four species (names in bold font) displayed a significantly different abundance in the exposure and control groups (adjusted *P* < 0.25) after adjusting for all feeding types using a linear regression model in the MaAsLin2 R package. (C) Relative abundance of the four differentially abundant bacterial species in the exposure and control groups: (left to right) *Bacteroides vulgatus*, *Bifidobacterium pseudocatenulatum*, *Clostridium neonatale*, and *Clostridium innocuum*.

We analyzed species-level differences in the gut microbiota in the exposure and control groups. Although there were some unique bacterial species in each feeding type that only appeared in either the exposure group or the control group, the species identified in both groups accounted for most sequencing reads (Fig. S5). This suggested that stool samples from both groups largely contained the same bacterial species. Next, we focused on species with a prevalence greater than 50% in each feeding type and compared their abundances in the exposure and control groups. Results showed that *Clostridium neonatale* had a significantly lower prevalence in the exposure group than in the control group among infants with mixed feeding (*P* = 0.04, Fig. 2A). Other prevalent species did not significantly differ in abundance between the exposure and control groups. We ranked the bacterial species based on their abundance (sequencing reads) across all 63 samples and identified the top 44 most abundant species that accounted for more than 90% of all sequencing reads. The relative abundance of these 44 species was standardized and plotted for each sample (Fig. 2B). The following four species displayed different abundances (adjusted *P* < 0.25) between the exposure and control groups after adjusting for the feeding type, and all four were depleted in the exposure group compared to the control group (Fig. 2C): *Bacteroides vulgatus* (*P* = 0.02), *Clostridium innocuum* (*P* = 0.03), *Bifidobacterium pseudocatenulatum* (*P* = 0.04), and *Clostridium neonatale* (*P* = 0.04). The abundances of these four species displayed similar trends among each feeding type (Table S1).

### Comparison of the metabolic capacities

**FIG 3:**
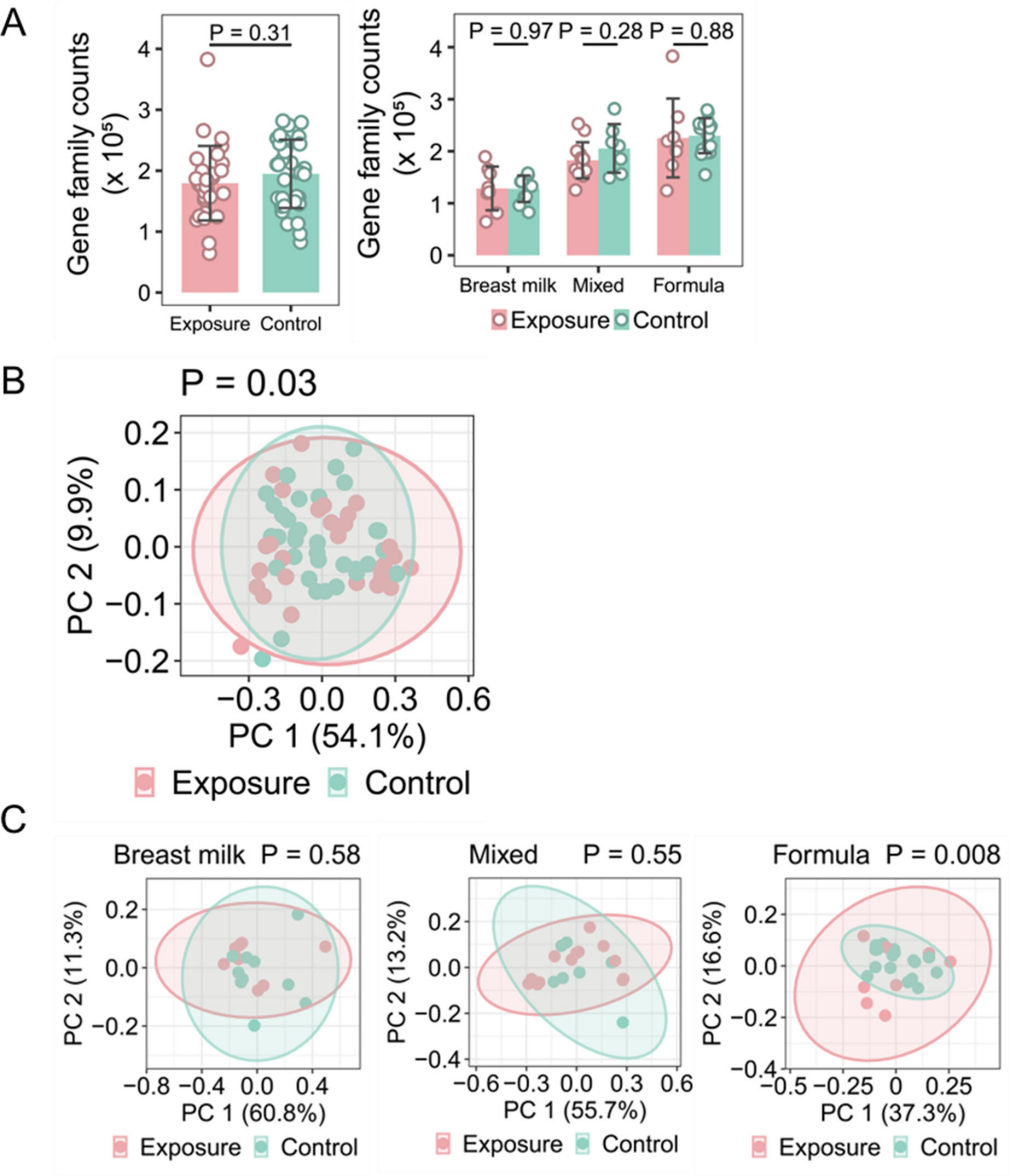
Metabolic gene family and compositional analysis between the exposure and control groups. (A) Metagenomic DNA sequencing counts of bacterial gene families in the exposure and control groups. Left, across all feeding types; right, subgroup analysis stratified by the feeding type. (B) PCoA plot of KEGG gene modules in the exposure and control groups. (C) PCoA plot of KEGG gene modules in the exposure and control groups stratified by the feeding type. The statistical significance of (A) was evaluated using the Student’s *t*-test. The statistical significance of (B) and (C) was evaluated using the analysis of similarities test. PC, principal coordinate.

Bacterial metabolites are believed to be an essential mechanism through which microbes affect the host (47). Therefore, we analyzed the metabolic potential of the gut microbial community based on microbial metagenomes. This functional profiling was performed by assigning DNA reads to metabolic gene families using HUMAnN3. We first assessed the overall gene families identified in the exposure group versus the control group, but we did not detect any significant differences (Fig. 3A). This result suggested that the total number of metabolic pathways in the samples from these two groups did not differ, although infants in the exposure group had significantly lower gut microbial diversity than those in the control group. Similar numbers of gene families were also identified in the exposure and control groups, irrespective of the feeding type (Fig. 3A).

Next, we compared the compositions of the KEGG modules between the exposure and control groups and detected a significant difference (*P* = 0.03), indicating that the infant gut metabolic potential in the exposure group may be altered compared to that in the control group (Fig. 3B). Subgroup analyses revealed that this difference was primarily due to significant differences in infants fed formula (*P* = 0.008, Fig. 3C), which was consistent with results from the microbial composition analysis. These combined results indicated that infants who were exposed *in utero* to Hurricane Maria and fed formula displayed significant alterations in their gut microbial community and metabolic capacity.

**FIG 4:**
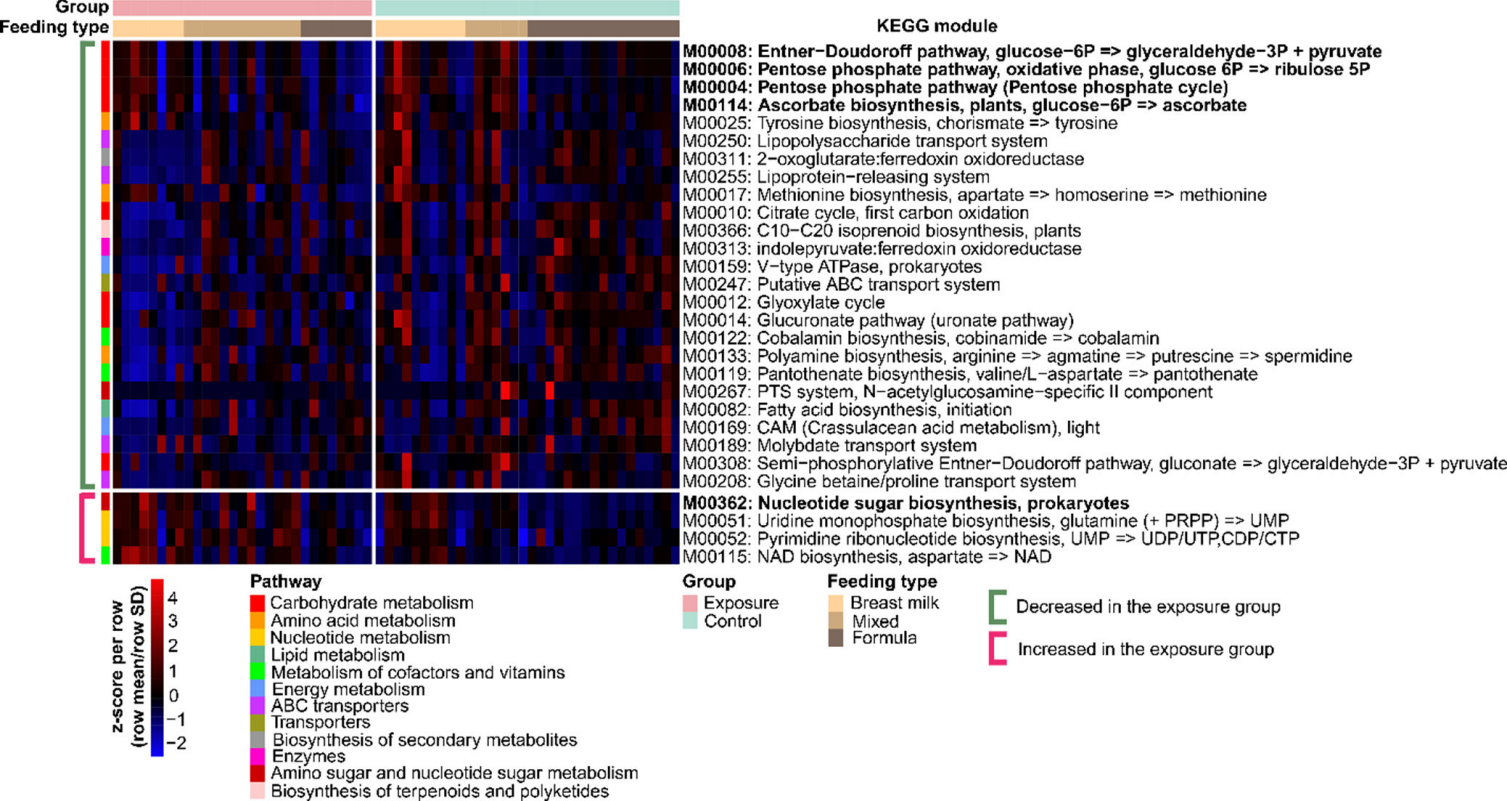
Heatmap of the *Z*-score of significantly altered metabolic modules in the exposure and control groups. Significantly altered metabolic modules were determined using a linear regression model in the MaAsLin2 package, with adjusted *P* < 0.25 as the cutoff for significance. Each column represents a sample grouped by its exposure status and feeding type and ordered by the infant age at sampling from youngest to oldest. Each row represents one significant metabolic module, grouped by a decrease or increase in the exposure group and ordered by the adjusted *p*-value from smallest to largest. Five modules with an adjusted *P* < 0.05 are bolded by name. Pathways that each module belongs to are indicated by color.

We identified 305 KEGG modules across all 63 samples. After adjusting for the feeding type, we identified 29 metabolic modules that were different between the exposure and control groups with an adjusted *P* < 0.25 (Fig. 4). Five out of the 29 modules significantly differed between the two groups (adjusted *P* < 0.05). These included four metabolic modules that were significantly lower in the exposure group and one metabolic module that was significantly higher in the exposure group than in the control group (Fig. S6). Metabolic modules that were significantly lower in the exposure group involved carbohydrate metabolism pathways, indicating that infants in the exposure group may have had a suppressed capacity to metabolize carbohydrates.

## DISCUSSION

Climate change has increased the frequency and intensity of hurricanes and floods (16, 48). Such climate events can have a profound impact on the human gut microbiome, although research is still scarce (49), and disproportionately affect the health outcomes of vulnerable populations, such as developing fetuses and infants (50, 51). This study analyzed the gut microbiome and metabolic capacity of infants who were exposed *in utero* to a devastating hurricane. Our results demonstrated a loss of microbial diversity and alterations in the gut microbiome composition and metabolic capacity in the stool of infants who were exposed to Hurricane Maria during their prenatal stage. Rich and diverse gut microbiota play a central role in the development and maturation of host innate and adaptive immunity (52 – 54). A loss of microbial biodiversity in the infant gut microbiome has been linked to increased risks of immune disorders (3, 55 – 57). Therefore, our results implied that microbial alterations may affect the health outcomes of hurricane-exposed infants, and evaluating their growth and health phenotypes is warranted.

We recently reported a significant increase in the microbial diversity and environmental or opportunistic pathogens in the nasal microbiome of exposed infants from our HOLA study (20), which was likely due to transient spikes in pathogens in post-storm environments (58 – 61). In contrast, the infant gut microbiome displayed decreased microbial diversity in exposed infants compared to the controls, suggesting that factors other than environmental exposures during the aftermath of Hurricane Maria specifically impacted the infant gut microbiome. Accumulating evidence indicates that prenatal maternal stress is associated with the offspring’s gut microbiome and drives inter-individual variation in the infant microbiome (28, 30, 62). It is possible that prenatal maternal stress due to the hurricane played a key role in influencing the offspring’s gut microbiome. It is worth noting that we did not find a statistical difference in maternal depression levels during sampling between the exposure and control groups. However, maternal depression that is evaluated at 2 to 6 months postpartum may not reflect the psychological stress that may have been experienced during the gestational stage. Further research is needed to better elucidate the important association between prenatal maternal stress due to a climate disaster and the offspring’s gut microbiome.

Four bacterial species, including *Bacteroides vulgatus*, *Bifidobacterium pseudocatenulatum*, *Clostridium innocuum*, and *Clostridium neonatale*, were found to have decreased relative abundance in the exposure group compared to the control group. *Bacteroides vulgatus* and *Bifidobacterium pseudocatenulatum* are both beneficial bacteria in nursing infants that modulate inflammatory responses and the gut metabolome (63 – 67). *Bifidobacterium pseudocatenulatum* also plays a regulatory role in mediating the gut-brain axis (68). Interestingly, a recent study found that maternal psychosocial stress during pregnancy was associated with a reduced abundance of *Bifidobacterium pseudocatenulatum* in the infant gut microbiome (69). *Clostridium innocuum* and *Clostridium neonatale* are both gut commensals, but they can also be potential pathobionts (70 – 73). A study found that a reduced abundance of *Clostridium neonatale* in the infant gut was associated with higher risks of preschool-age asthma, suggesting that *Clostridium neonatale* plays a positive role in developing the infant immune system (74).

In our study, gut microbial alterations associated with the prenatal disaster exposure were primarily observed in infants who were formula fed, suggesting that breastmilk, even if partially given to the offspring, may mitigate a disruption of gut microbial development due to a prenatal disaster exposure. Breastmilk is a well-adapted nutritional supply in the postnatal stage (75). Many bioactive substances shape the infant gut microbiome and metabolic networks by serving as substrates for specific microbes, such as human milk oligosaccharides, or interacting with microbes, such as maternal IgA antibodies and maternally derived cytokines (76 – 79). Our study supports a protective effect of breastfeeding on the infant gut microbiome, specifically during a natural disaster. Since breastfeeding practices are largely impacted by inadequate disaster responses and subsequent barriers and challenges that mothers face during disasters, further studies and interventions are needed to understand, protect, and support breastfeeding during natural disasters (80, 81). A previous study reported that gut microbial alterations in infants exposed to prenatal depression were dependent on breastfeeding, although alterations in infant gut immunity, such as impaired secretory IgA levels, were universal and independent of the feeding type (28). Therefore, additional studies are needed to further characterize the gut immunity of infants who are exposed *in utero* to a disaster.

Our results suggested that prenatal disaster exposures may be associated with reduced metabolic capacity in the gut microbial community. Four gene modules in carbohydrate metabolism were significantly lower in the exposure group than in the control group. Carbohydrates are important nutrients that support health and immunity (82), and a reduced carbohydrate metabolism is an indicator of gut microbial disturbance, such as during a norovirus infection (83). Our study underscored the importance of investigating the effects of prenatal disaster exposures on infant gut metabolic pathways in addition to their microbial composition.

This study had several limitations. (i) Our HOLA study was established in a post-disaster setting, so recruitment was difficult and the number of participants was relatively small. However, we would like to highlight the geographic, ethnic, and medical homogeneity of our cohort. The recruitment was restricted to Hispanic Islander infants who lived in the metropolitan San Juan region, were full-term and vaginally born, and were not recently on antibiotics or any other medications. (ii) The control and exposure groups may have some potential differences that led to the microbial differences. We attempted to limit the factors that may cause such differences. For example, we recruited the exposure and control groups from the same metropolitan region of San Juan during the same season in 2018 and 2019, respectively. We also used the same approaches to collect and process the samples from both groups. In addition, we performed shotgun metagenomic sequencing on all the samples at the same time. However, there was a specific concern about differences in the drinking water that was used to prepare baby formula between the two groups. Although a study characterizing the microbial composition of drinking water following Hurricane Maria across nine drinking water systems in Puerto Rico found a relatively stable microbial concentration based on metagenomic sequencing, drinking water may still be a potential factor that caused the differences in the infant gut microbiome in the formula feeding group (61). Future studies should collect more information from families about their drinking water source and collect water samples as well. (iii) There is a possibility that the observed differences in the infant gut microbiome were caused by differences in exposures at the postnatal stage or by a mixed influence at both the pre- and postnatal stages. The majority of adverse experiences reported by the mothers in the exposure group occurred during their gestational stage. Major public works, housing, and healthcare had been restored during our first recruitment period (March 2018 to August 2018). Therefore, we believe that the marked differences between the exposure and control groups were within the prenatal maternal stage. However, Hurricane Maria may have had other long-lasting implications, and it is reasonable that there were still differences in the postnatal stage between the two groups that impacted the infant gut microbiome. (iv) Information on potential confounders, such as maternal obesity, pregnancy complications, excess gestational weight gain, hyperglycemia, maternal antibiotic use during the third trimester and lactation, and neonatal birth weight, that may have influenced the infant gut microbiome was not collected from our questionnaires, so we could not perform analyses on these factors and rule out their influence on our results. However, future studies should consider characterizing more potential influencing factors of the infant gut microbiome.

### Conclusion

In conclusion, HOLA was a pioneering study that assessed the gut microbiome of infants who were exposed *in utero* to Hurricane Maria, a devastating disaster in Puerto Rico. We compared the gut microbiome of infants who were *in utero* when Hurricane Maria struck Puerto Rico with that of infants who were conceived at least 5 months afterward. Our results showed that infants who were exposed to Hurricane Maria during gestation had reduced microbial diversity, and breastfeeding can adjust the composition of the gut microbiomes of exposed infants. These results add valuable insights into the health impacts of extreme weather events and climate change on vulnerable populations. Our HOLA study indicated that alterations in the infant gut microbiome could be a mechanism underlying long-term disease risks in offspring exposed *in utero* to disasters. Strategies are needed to identify infants with altered gut microbiomes, particularly infants who were exclusively formula-fed. This and future studies will provide opportunities to implement early interventions to reduce negative long-term health outcomes in populations that had disaster exposures during their prenatal stage.

## Data Availability

The raw metagenomic sequencing data generated in this study have been deposited in the Sequence Read Archive (SRA) under the BioProject accession number PRJNA938144.

## SUPPLEMENTAL MATERIAL

Supplemental material msphere.00134-23-s0001.docx Supplemental table and figures.

ASM does not own the copyrights to Supplemental Material that may be linked to, or accessed through, an article. The authors have granted ASM a non-exclusive, world-wide license to publish the Supplemental Material files. Please contact the corresponding author directly for reuse.
