# Supplementary material for: Infants exposed in utero to Hurricane Maria have gut microbiomes with reduced diversity and altered metabolic capacity
Source: mSphere. 2023 Sep 27;8(5):e00134-23. doi: 10.1128/msphere.00134-23 (PMC10597457; doi:10.1128/msphere.00134-23)
Supplement: Supplemental material — Supplemental table and figures. [file msphere.00134-23-s0001.docx]

**Supplementary**

**Infants exposed *in utero* to Hurricane Maria have gut microbiomes with reduced diversity and altered metabolic capacity**

Ai Zhang^1^, David de Ángel Solá^2^, Midnela Acevedo Flores^3^, Lijuan Cao^1^, Leran Wang^4^, Josh G. Kim^1^, Phillip I. Tarr^5^, Barbara B. Warner^5^, Nicolás Rosario Matos^3^, Leyao Wang^1,*^

^1^ Department of Medicine, Division of Allergy and Immunology, Washington University School of Medicine in St. Louis, St. Louis, Missouri, U.S.

^2^ Department of Pediatrics, Yale School of Medicine, New Haven, Connecticut, U.S.

^3^ San Juan City Hospital Research Unit, Department of Pediatrics and Obstetrics and Gynecology, San Juan Hospital, San Juan, Puerto Rico

^4^ Department of Medicine, Division of Infectious Diseases, Edison Family Center for Genome Sciences and Systems Biology, Washington University School of Medicine in St. Louis, St. Louis, Missouri, U.S.

^5^ Department of Pediatrics, Washington University School of Medicine in St. Louis, St. Louis, Missouri, U.S.

*** Corresponding author:** Leyao Wang, PhD, MPH

Division of Allergy and Immunology, Department of Medicine, Washington University School of Medicine in St. Louis.

425 South Euclid Avenue, Campus Box: 8122. St. Louis, Missouri, U.S. 63110

Email: [Leyao.wang@wustl.edu](mailto:Leyao.wang@wustl.edu)

**Supplementary Table S1 | Relative abundance (%) of four bacterial species that were differently abundant by group.**

| **Bacterial species** | **Exposure group** | **Control group** | ***p*** |
| --- | --- | --- | --- |
|  | **Median (IQR)** | **Median (IQR)** |  |
| ***Bacteroides vulgatus*** |  | |  |
| Breastfeeding | 0.006 (0, 0.289) | 0.695 (0, 9.021) | 0.38 |
| Mixed | 1.616 (0, 11.955) | 6.31 (1.35, 18.094) | 0.26 |
| Formula | 0.879 (0, 7.641) | 5.211 (2.165, 9.093) | 0.28 |
| ***Bifidobacterium pseudocatenulatum*** |  |  |  |
| Breastfeeding | 0 (0, 0.032) | 0 (0, 0) | 1 |
| Mixed | 0 (0, 0) | 0 (0, 15.08) | 0.28 |
| Formula | 0 (0, 0.011) | 4.137 (0, 12.189) | 0.09 |
| ***Clostridium neonatale*** |  |  |  |
| Breastfeeding | 0 (0, 0.004) | 0.012 (0, 0.899) | 0.24 |
| Mixed | 0 (0, 0.063) | 0.23 (0.078, 0.329) | 0.09 |
| Formula | 0.092 (0, 0.368) | 0.013 (0.001, 0.087) | 0.7 |
| ***Clostridium innocuum*** |  |  |  |
| Breastfeeding | 0 (0, 0) | 0 (0, 0.149) | 0.06 |
| Mixed | 0 (0, 0) | 0.006 (0.003, 0.059) | 0.21 |
| Formula | 0.032 (0, 0.086) | 0.23 (0.037, 0.555) | 0.13 |
| Significance was evaluated based on the Wilcoxon rank sum test. IQR, interquartile range. | | | |


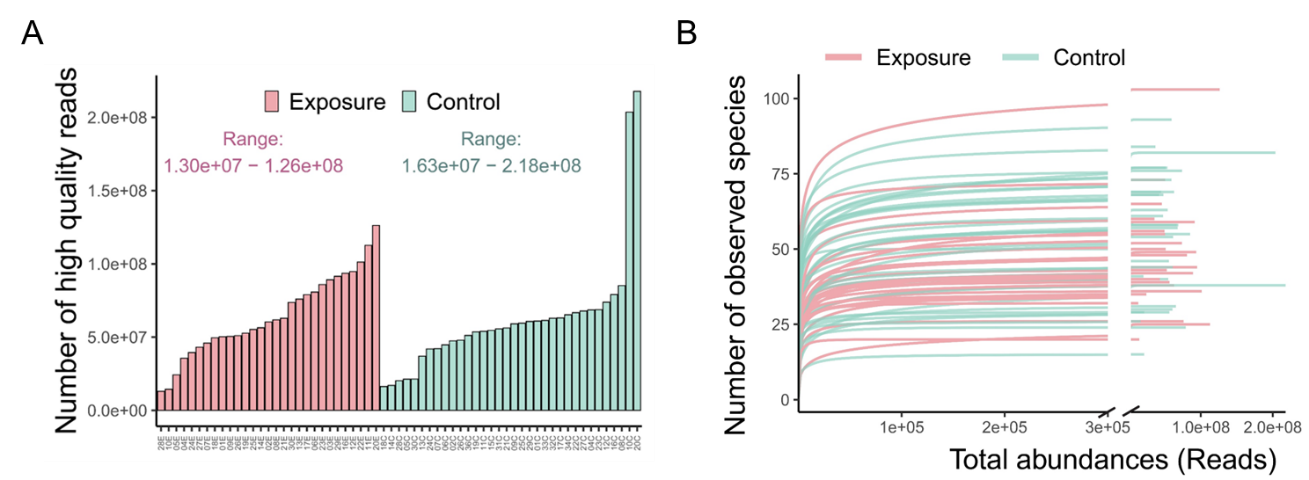


**Supplementary Figure S1 | Number of sequencing reads and the species richness of each sample.** **A**, Number of quality reads of shotgun metagenomic sequencing for each stool swab sample after quality control procedures. **B**, Rarefaction curve of richness against sequencing reads.

**
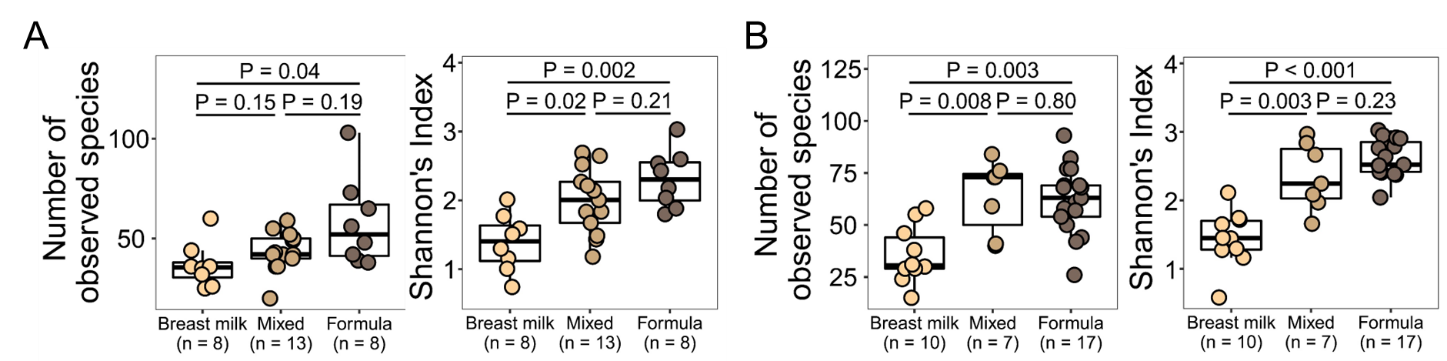
**

**Supplementary Figure S2 | Analysis of α-diversity for the number of observed species and the Shannon index by the feeding type.** **A**, Exposure group. **B**, Control group.

**
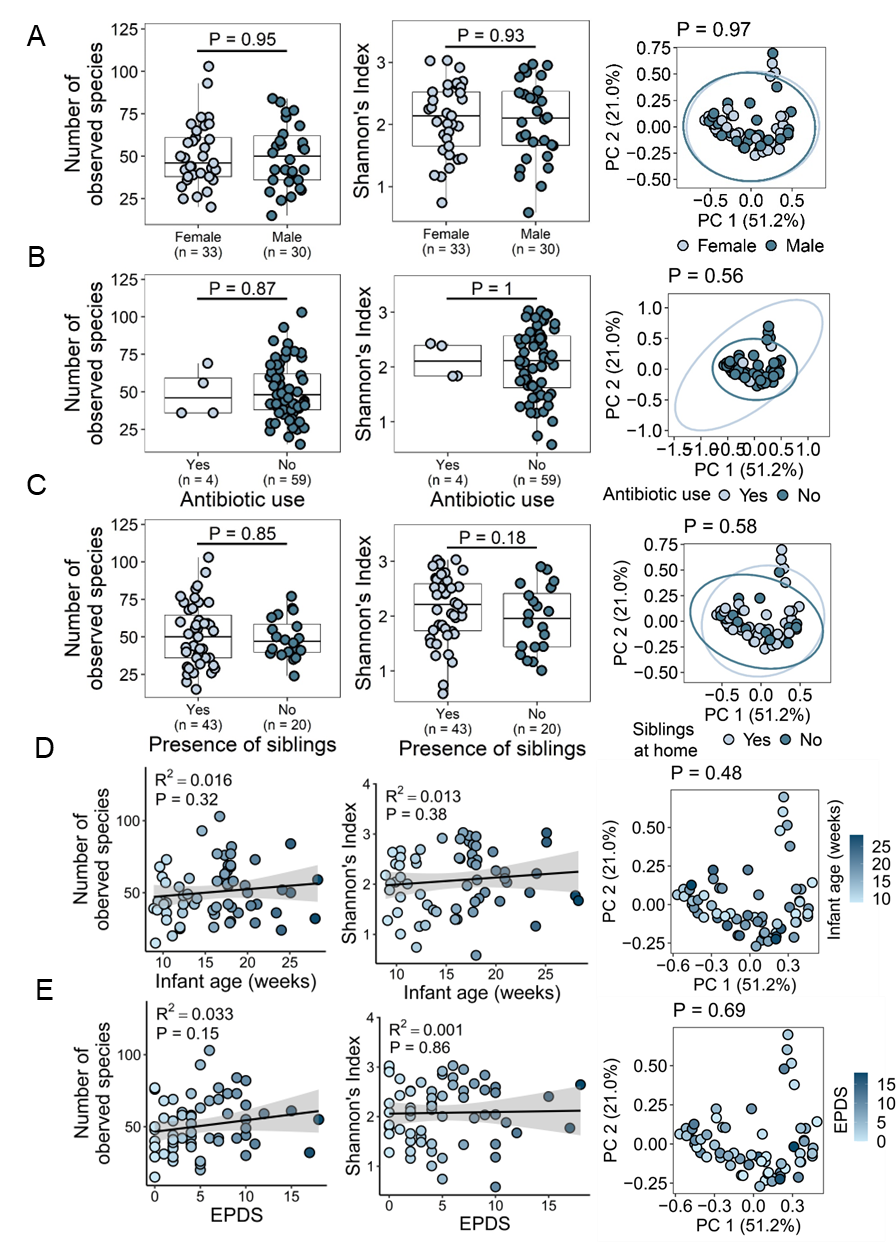
**

**Supplementary Figure S3 | Comparisons of the gut microbiome by different factors for α-diversity (right and middle plots) and β-diversity (PCoA plot based on weighted UniFrac distances, left plots).** **A**, Sex. **B**, Antibiotic use in the previous four weeks. **C**, Presence of siblings at home. **D**, Infant age at sampling. **E,** Maternal Edinburgh Postnatal Depression Scale (EPDS) score at sampling. Statistical significance of the α-diversity analyses was based on the Wilcoxon rank sum test. Statistical significance of the β-diversity was determined using the analysis of similarities (ANOSIM) test. PC, principal coordinate.


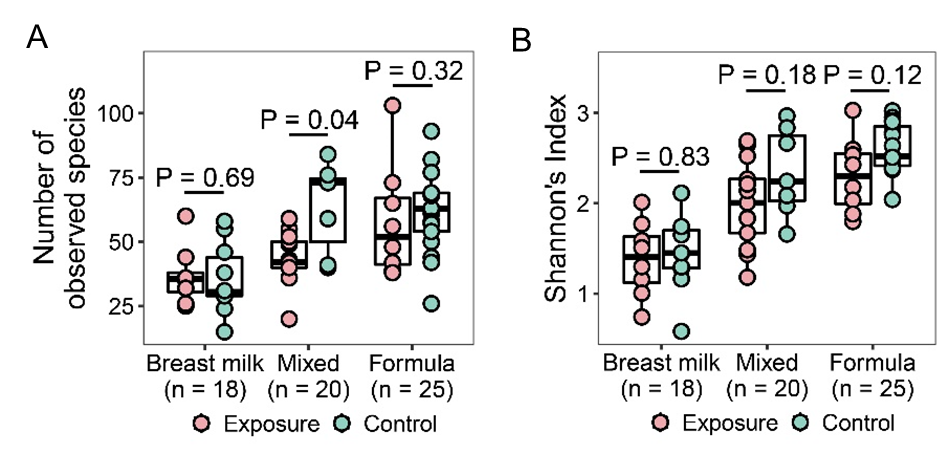


**Supplementary Figure S4 | Analysis of α diversity for each feeding type.** **A**, Number of observed species. **B**, Shannon index.

**
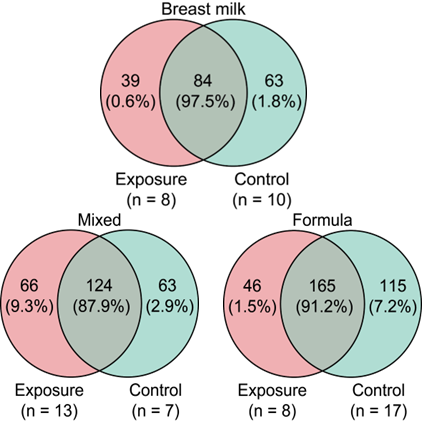
**

**Supplementary Figure S5 | Number and percentage of reads for species detected in only one group or both groups according to the feeding type.**

**
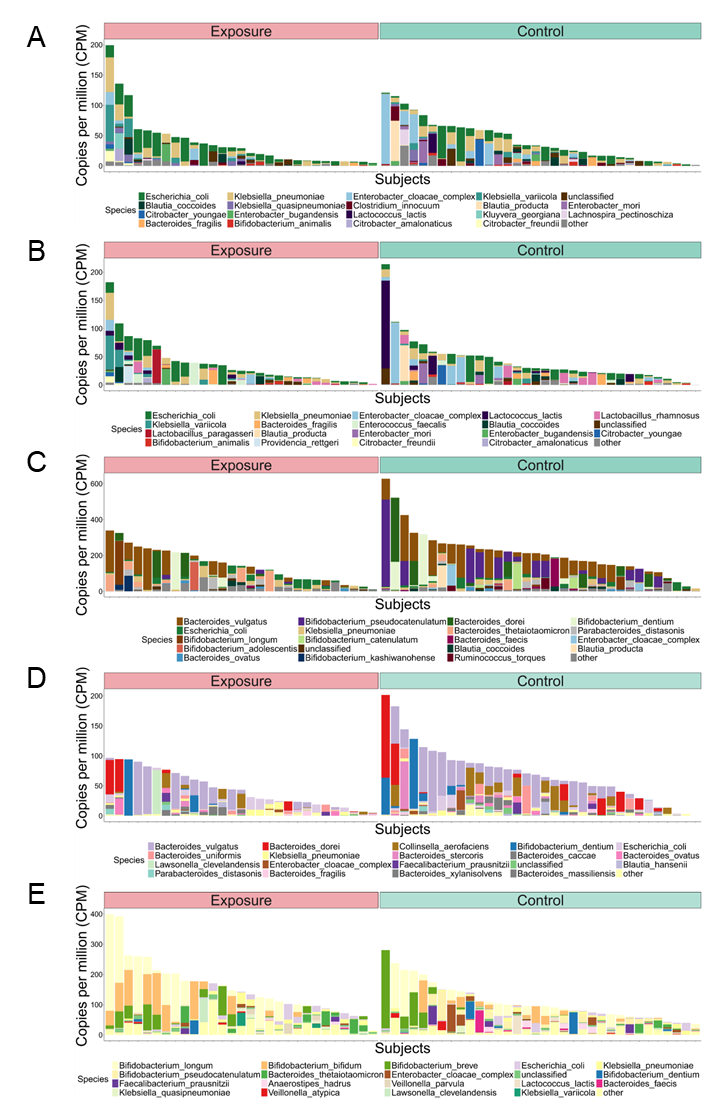
**

**Supplementary Figure S6 | Bacterial species that contributed to each of the five significantly different metabolic modules detected in the exposure group.** Each bar represents a sample grouped by its exposure status and ordered by the abundance of each metabolic module. **A**, M00008: Entner-Doudoroff pathway; glucose-6P → glyceraldehyde-3P + pyruvate. **B**, M00006: Pentose phosphate pathway, oxidative phase; glucose 6P → ribulose 5P. **C**, M00004: Pentose phosphate pathway (pentose phosphate cycle). **D**, M00114: Ascorbate biosynthesis, plants; glucose-6P → ascorbate. **E**, M00362: Nucleotide sugar biosynthesis, prokaryotes.
